# Aggregate Formation of Boron-Containing Molecules in Thermal Vacuum Deposited Films

**DOI:** 10.3390/ma14195615

**Published:** 2021-09-27

**Authors:** Oleksandr Navozenko, Valeriy Yashchuk, Oleksiy Kachkovsky, Dalius Gudeika, Rita Butkute, Yuriy Slominskii, Volodymyr Azovskyi

**Affiliations:** 1 Faculty of Physics, Taras Shevchenko National University of Kyiv, 64/13 Volodymyrs’ka Str., 01601 Kyiv, Ukraine; yashchukvaleriy@gmail.com (V.Y.); vladimirazovskij@gmail.com (V.A.); 2 V.P. Kukhar Institute of Bioorganic Chemistry and Petrochemistry, Murmanskaya Street, 1, 02660 Kyiv, Ukraine; adkachkovsky@gmail.com; 3 Department of Polymer Chemistry and Technology, Kaunas University of Technology, Radvilenu pl. 19, LT-50254 Kaunas, Lithuania; rita.butkute@ktu.lt; 4 Institute of Organic Chemistry, National Academy of Sciences of Ukraine, 5 Murmanska Str., 02660 Kyiv, Ukraine; yuriy.slominskii@gmail.com

**Keywords:** fluorescence, dimers, quantum chemical modeling, thermal vacuum deposition, cyanine dyes, boron-containing molecules, in situ spectra

## Abstract

The spectral properties of new boron-containing dyes were studied. One-component (pure dyes) and composite “Alq_3_+dye” thin films were fabricated using the thermal vacuum deposition method. The positions of the transmission spectra maxima in a one-component film are different for different film thicknesses. The best correlation of the maxima positions of the dye transmission spectra in solid and liquid solutions was observed for thicknesses of films close to a few (up to 10) monolayers. On the other hand, the absorption spectra maxima positions of one-component dye films (upper 10 nm) and composite films with high concentration, did not match the corresponding positions of absorption spectra maxima recorded in solutions. Comparison of the absorption spectra in one-component dye films and in solutions indicates the presence of both monomers and their aggregates in one-component films (contrary to solutions where such processes of aggregation do not take place, even at very high concentrations). Simultaneously with aggregation manifestation in the absorption spectra, the intensity of fluorescence of one-component dye films dramatically decreases. A quantum chemical simulation of the possible relative arrangement of two dye molecules indicates that the most possible of the simplest types of aggregates are physical dimers. Films of practical importance (due to efficient energy transfer from host to guest molecules when all singlet excitons are captured) possess a high quantum yield of fluorescence when reaching an impurity concentration of a few percent (aggregation does not take place yet).

## 1. Introduction

In recent decades, researchers have been particularly interested in the development of new, more effective organic devices, for example, OLEDs, OFETs, etc., [1]. Boron-containing dyes can give a perspective for these purposes due to their high molar absorption coefficient (40,000 to 110,000 M^−1^ cm^−1^), high fluorescent quantum yield (60–90%), relatively long fluorescence lifetime (from 1 to 10 ns), superior charge-transfer properties and excellent stability in solution as well as in solid states [2,3]. Typically, for such types of dyes the negligible relaxation in the excited state takes place and, thus, very small Stokes shifts are observed [4]. Such organic chromophores are molecules with spatially separated donor–acceptor terminal groups. In such systems, the absorption region can be varied by changing the chemical structure of the terminal groups. Based on such types of dyes, composite thin films can be thermally deposited in a vacuum with host and guest molecules [4]. However, cyanine dyes form aggregates of different types under some conditions. Dyes can aggregate when the distance between molecules is rather small under some circumstances. This is achieved usually in highly concentrated solutions. In thin one-component cyanine films (solid) distances between molecules are small compared with the distances between them in liquid solutions; therefore, aggregates can be formed in one-component films with higher probability [2]. Moreover, one molecule with another can form aggregates of a certain type with a special spatial geometry, depending on their chemical structure and the relief of the substrates [5]. Dye aggregation is also solvent dependent and temperature dependent [6]. It is known that the rigid, planar geometry of some boron-containing chromophores (BODIPY) also leads to a propensity to aggregate through π–π stacking [7]. In the case of nonsymmetric boron-containing chromophores, the formation of physical dimers is also observed. In this case, contrary to BODIPY chromophores, boron-containing dyes possess high values of state dipole moments. The presence of aggregates, in turn, leads to both an increasing and a decreasing luminescence quantum yield. The formation of J-aggregates leads in a number of cases to aggregation-induced emission (AIE); the formation of H-aggregates, on the contrary, leads to aggregation-caused quenching [8,9]. The absence of aggregates in a thin film can be achieved by decreasing of the molecules impurity concentration in the matrix medium. This prevents direct interchromophore interactions, which in some cases is important because rapid excited-state quenching results in a substantial reduction in the PL quantum yield [7].

For the dyes under study, it is convenient to select Alq_3_ as a matrix because its absorption band belongs to the first electronic transition and it is located at a shorter wavelength than the corresponding band of the dye. That is why the emission spectra of a matrix and dyes do not overlap, which is convenient for a design of emitting layers. Our investigation proves that under some circumstances of a thermal vacuum deposition of dyes the absorption band of film appears that can be associated with physical dimers or aggregate formation. It should be emphasized that such types of physical dimers do not manifest in solutions of these compounds. This interesting property, the quantum calculation of possible pre-dimer configurations and the general photophysical properties of pure Alq_3_ and “Alq_3_+dye” films (thermally deposited in a vacuum), is discussed in the presented paper.

## 2. Materials and Methods

**Figure 1:**
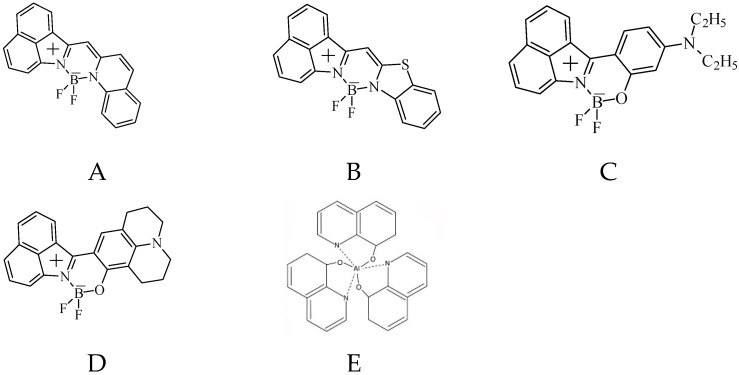
The chemical structures of guest dyes (I (**A**), II (**B**), III (**C**), IV (**D**)), and Alq_3_ (**E**) matrix.

The structures of guest dyes I, II, III, IV, and Alq_3_ matrix are presented in Figure 1.

Thin films were grown by thermal vacuum deposition method on glass and quartz substrates under the pressure of the order 1 × 10^−5^ Pa. Composite films were fabricated through co-evaporation of dye (dopant) together with Alq_3_ (matrix). Each composite thin film consists of matrix substance (Alq_3_) and also contains one dye (I, II, III, IV) as impurity. Fluorescence spectra were registered on spectrofluorometer Carry Eclipse (Varian), absorption spectra were registered on Specord UV Vis. In situ (during the process of deposition) spectra of dyes layers were recorded using a matrix fiber optic spectrometer Polytec StellarNet EPP. To study the processes of aggregate formation, a weakly polar solvent of toluene was used, which does not affect the ability of molecules to aggregate. The highly polar solvent tetrahydrofuran was used for comparison. All calculations were performed using Gaussian 16 package [10]. Molecular geometries in ground state of the single molecules and pre-dimeric configurations were optimized within static DFT electronic structure theory coupled with WB97XD functional (basis set 6,31 G (d,p)). Spectrum calculations utilized TD-DFT approach and WB97XD exchange—correlation functional (basis set 6,31 G (d,p)) [11].

## 3. Results and Discussion

We investigated the aggregation of new boron-containing dyes using the measurements of absorption, fluorescence, and excitation of the fluorescence spectra of solutions (the in situ transmission spectra of films of different thickness were recorded in real time during the deposition process), and the results of the quantum chemical parameter calculations of the high electronic states of the dyes’ monomeric molecules and their pre-dimeric states. In a previous paper [12], it was shown that these dyes possess a high quantum yield in liquid solutions, but quantum yields in thin films (one-component) obtained by thermal vacuum deposition are rather low, contrary to liquid solutions. The same effect is observed for two-component films (“Alq_3_-matrix + guest dyes”). This is undesirable for the application of these dyes in luminescent materials. For the study of this problem, the formation of supramolecular structures (including of the in situ spectra recorded during the deposition process) was investigated.

### 3.1. Spectral Properties of Components of Matrix (Alq_3_) and Impurities (Guest Dyes)

**Figure 2:**
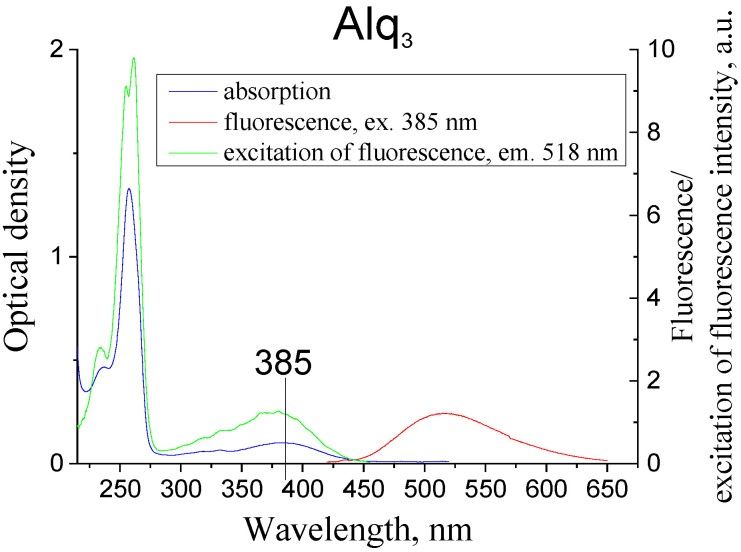
Absorption, fluorescence, excitation of fluorescence of Alq_3_ in acetonitrile solution (C = 6.7×10^−6^ g/cm^3^, T = 293 K).

The absorption, fluorescence, and fluorescenсe excitation spectra of Alq_3_ in solution are shown in Figure 2.

**Figure 3:**
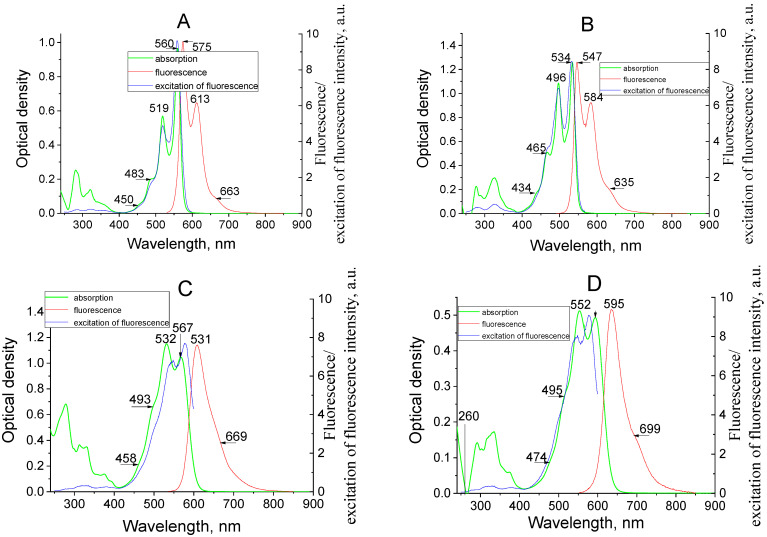
Absorption, fluorescence, and excitation of fluorescence spectra of dyes I (**A**), II (**B**), III (**C**), IV(**D**) in tetrahydrofuran (concentration 1 × 10^−6^ g/сm^3^, T = 293 K).

The absorption and fluorescence spectra of the dyes’ molecules in tetrahydrofuran solutions are shown in Figure 3. The calculated characteristics of the lowest electron transitions are collected in Table 1. In the longwave region (400–600 nm) of the absorption spectra the comparatively intensive band is observed; the next shortwave wide bands are located in the spectral region <400 nm. As it follows from Table 1, the energies of the higher transitions are essential higher and dipole moments are significantly (at the order) lower in comparison with the first electronic transition. Thus, the longwave spectral band corresponds, undoubtedly, to the first electron transition (see Figure 3), while the wide bands in the shortwave spectral region are connected with the next transitions.

The first electronic transitions in all molecules involves, practically, only two frontier orbitals: the highest occupied molecular orbital (HOMO) and the lowest unoccupied MO (LUMO); their transition dipole moments are high enough in comparison with the next shortwave transitions (see Table 1). In contrast to the first electronic transition, the higher transitions involve the MO orbitals, which are close to HOMO–LUMO occupied or vacant MOs (HOMO-1; LUMO+1 etc.). The intensive spectral peaks in the absorption spectra correspond to the vibrational transitions. The appreciable divergence between the calculated and experimental wavelengths is a typical disadvantage of the DFT method [13]; however, it is enough to analyze correctly the nature of the electronic transitions.

The fluorescence spectra (in energy units) are symmetrical to the absorption spectra of dyes. This indicates that the same centers are responsible for absorption and emission. The Stock’s shifts in the fluorescence spectra are comparatively small; this points to the little geometrical changes upon excitation. 

**Figure 4:**
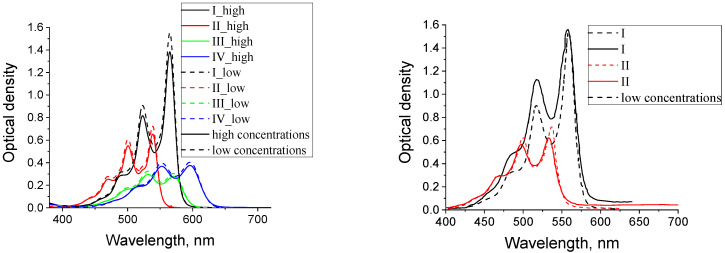
Dye absorption spectra I (C_high_=1.46 × 10^−4^ mol/L; C_low_=1.46 × 10^−5^ mol/L), II (C_high_=9.56 × 10^−5^ mol/L; C_low_=9.56 × 10^−6^ mol/L), III (C_high_=9.14 × 10^−5^ mol/L; C_low_=9.14 × 10^−6^ mol/L), IV (C_high_=8.58 × 10^−5^ mol/L; C_low_=8.58 × 10^−6^ mol/L) in toluene (**left**) and tetrahydrofuran (**right**). T = 293 K.

It is known that the best ability of molecules to aggregate is manifested in the neutral organic solvents [14]. Taking this into account, the absorption spectra of dye solutions in a practically non-polar solvent of toluene (ε = 2.3) were compared with the absorption spectra in polar tetrahydrofuran (ε = 7.6) at different concentrations (see Figure 4).

The similarity in the shape and correspondence of the spectral position of the dye absorption bands for high and low concentrations are clearly visible. Thus, we can conclude that the aggregation of these types of molecules in solutions does not occur. It is possible that aggregates are not formed because the critical concentration has not been reached at which the dye molecules would come close enough. Achieving significantly higher concentrations than are indicated in the caption to Figure 4 was not possible due to the poor solubility of the dyes.

### 3.2. Spectral Properties of Films. Aggregation in Studied Systems

**Figure 5:**
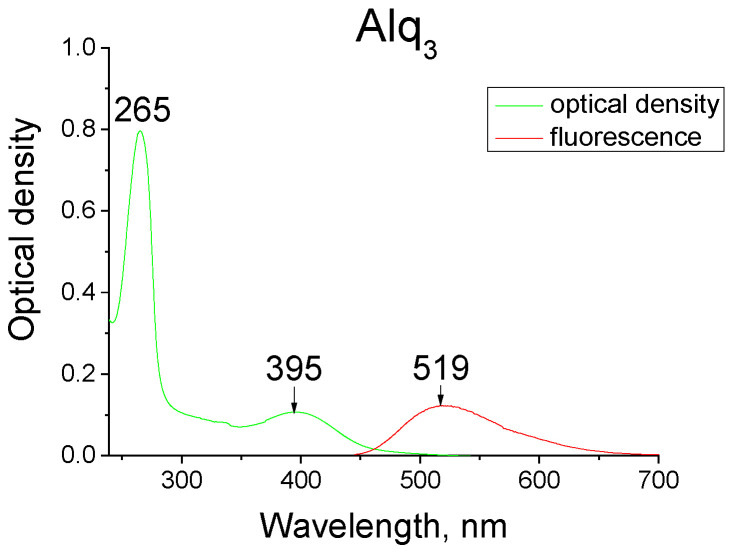
Absorption and fluorescence spectra of Alq_3_ in solid films (thickness is several hundred nanometers).

The absorption and fluorescence spectra of pure Alq_3_ in one-component solid films are shown in Figure 5. The presented data are in close agreement with other investigations [15,16].

**Figure 6:**
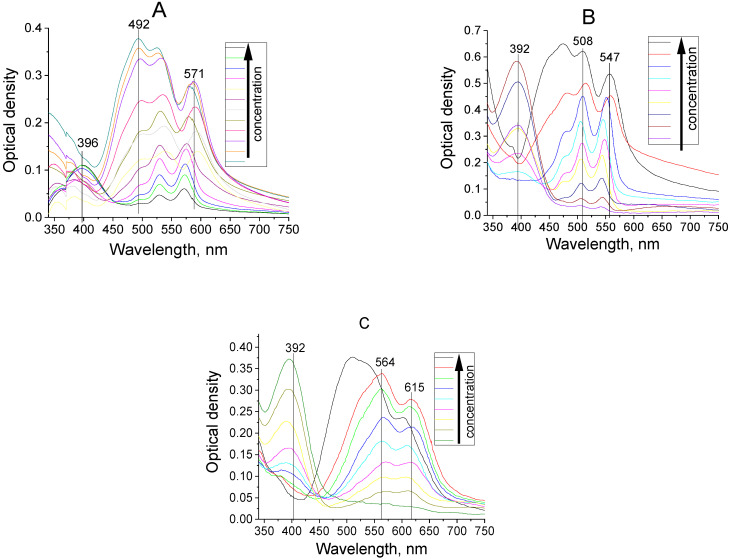
Absorption spectra of Alq_3_ composite films with different concentrations of guest dyes I (**A**), II (**B**), IV (**C**).

Figure 6 shows the absorption spectra of two-component films “Alq_3_+dye”. The absorption spectra of thin composite films at a low concentration of impurity molecules are close to the shape of the absorption spectra of solutions. Shortwave broad band appears with increasing concentration of the dye in Alq_3_ matrix.

### 3.3. One-Component Thin Dye Films (Case of High Concentrations)

**Figure 7:**
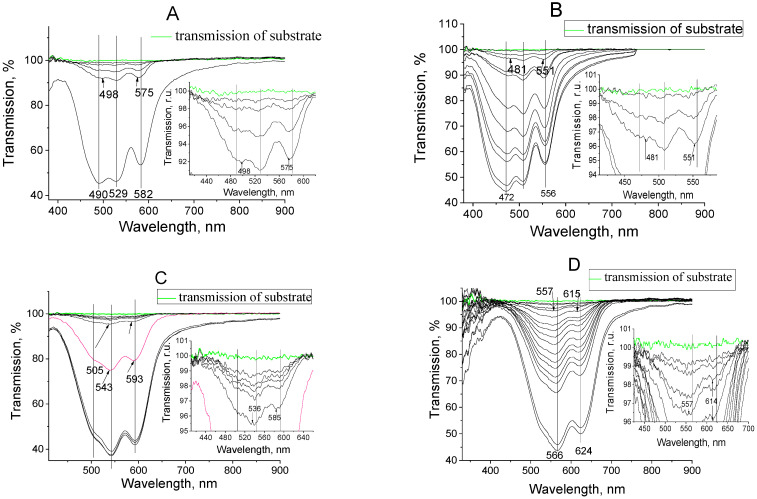
Absorption spectra of one-component dye films (I (**A**), II (**B**), III (**C**), IV (**D**)) registered at the time of their formation (in situ) in a vacuum chamber with an interval from one to 100 s of deposition. The glass substrates were used.

It was shown above that the absorption spectra of I, II, III, and IV dyes in a low-polar solvent of toluene at high concentrations (see Figure 4) are the same as in low concentrations. However, in some cases [17] (often in the case of neutral molecules), cyanine’s may form aggregates during thermal deposition in a vacuum since, in this case, they approach the smaller distances between themselves more than in solutions. Therefore, the transmission spectra of dyes in solid films (obtained in situ during their fabrication by the method of thermal vacuum deposition) were studied (see Figure 7). It is important here to compare the transmission spectra of germination films at the start of deposition (with a transmittance of 99.5–100%) and the spectra of the film with a thickness of about several hundred nanometers. The transmission spectra of several film monolayers do not significantly differ from the spectra of the dye solutions. Indeed, the positions of the minima of the transmission bands of several monolayers for all dyes are near to the corresponding bands in the liquid solution (see Figure 3, Figure 4, and Figure 7). The distribution of the values of transparences in spectra is close to the corresponding distribution for solutions too. The shortwave minimum transmission spectrum becomes more intensive than the minimums of the monomer bands with increasing thickness, and it becomes significantly more intensive than the minimum transmission spectrum of several layers. This increase is significant for dyes I and II, and for dyes III and IV since, as noted above, in this region, the bands are associated only with the first molecular electron transition. On the other hand, the observed spectral structure gives the evidence of the presence of some other centers of absorption in the films. It is known that the absorption and fluorescence bands diminish, and fluorescence and absorption spectra are shifted to shorter wavelengths with growing amounts of monomeric units of boron-containing dyes. This can be explained by the formation of other optical centers [18]. These studies lead to the conclusion that this type of spectral behavior is connected with dimers’ formation.

**Figure 8:**
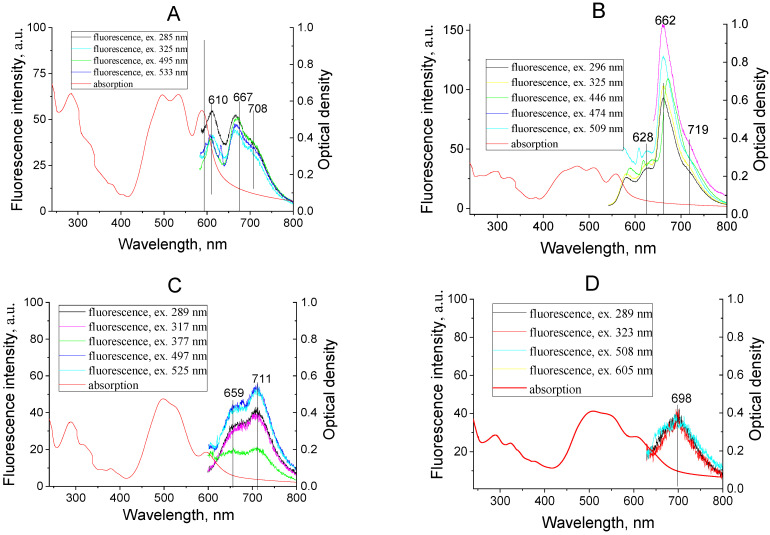
Absorption and fluorescence of one-component dye films I (**A**), II (**B**), III (**C**), IV (**D**), fabricated by thermal vacuum deposition.

Another confirmation of the dimers’ formation is the presence of the characteristic fluorescence of films, which differ from the fluorescence of the compounds in solutions. Generally, the fluorescence spectra of dimers are structureless and characterized by low intensity broad bands. The absorption and fluorescence spectra of one-component dye films are presented in Figure 8. In the absorption and fluorescence spectra of films, the vibronic structure of dye molecules is manifested on the ground of the strong aggregate band. In our opinion, the absence of a shortwave fluorescence band for films that correspond with the fluorescence first waveband for dye solutions is connected with reabsorption.

It should be noted that the vibronic structure in the absorption and fluorescence spectra of films compared to the spectra of solutions becomes more pronounced. Interaction between the molecules in the studied films manifests in the asymmetry of the monomeric absorption and fluorescence bands, as well as in a shift in the absorption and fluorescence spectra of the investigated films relative to the corresponding spectra of solutions.

**Figure 9:**
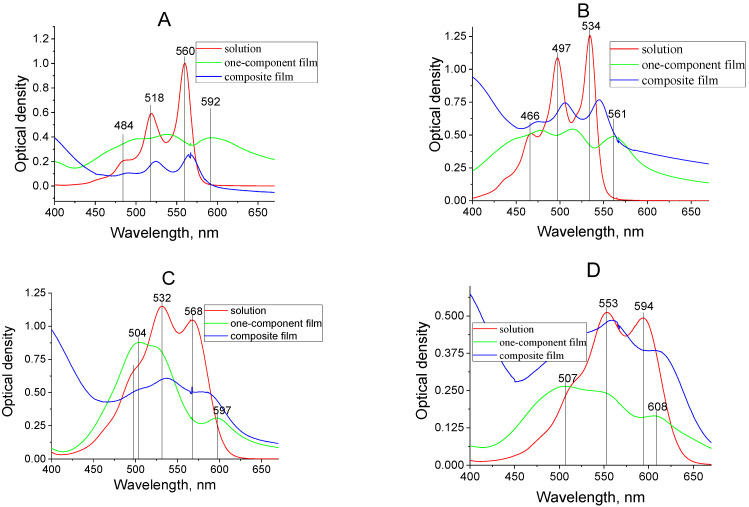
Absorption spectra of one-component, composite “Alq_3_+dye” films and tetrahydrofuran solutions of dyes I (**A**), II (**B**), III (**C**), IV (**D**).

In general, the structure of the absorption spectra of solutions and films of dyes I, II, III, and IV are close. This similarity indicates that, mainly, the dye molecules are involved in the film formation and there is no spectral manifestation of their fragments (any destruction processes or chemical modification of dye molecules due to the thermal deposition method do not happen). The recorded absorption spectra of one-component films of all dye samples were compared with the absorption spectra of dyes in solution and in the solid matrix Alq_3_. The results are presented in Figure 9. The comparison of the absorption spectra of dye molecules in tetrahydrofuran solution and Alq_3_ solid films shows that the positions and shapes of the bands are close only when the impurity concentration in the Alq_3_ matrix is 1–3 percent or less. In one-component dye films’ absorption spectra, the characteristic shortwave bands were revealed. A significant broadening of the absorption bands in films with a high concentration of impurity molecules is noticeable. Although it is known that cationic polymethine dyes derived from benzene [c, d] indole do not form physical dimers or aggregates in solutions, even at high concentrations, in a one-component film the situation may be different [14]. Moreover, the studied dyes are neutral derivatives of cationic dyes. Given this fact, as well as the fact that the studied molecules are almost flat, we can suggest that the possibility of the formation of physical dimers for this type of dye is high enough.

As can be seen from Figure 6, the shortwave dimer-band intensity increases with increasing impurity concentration in the Alq_3_ matrix, which is not observed in composite films at low concentrations of dye. Therefore, in our opinion, the most probable explanation for the difference in the absorption spectra of dyes in solutions and one-component films is the presence of both monomers and dimers in the films.

### 3.4. Quantum Chemical Studies of Configurations of the Spatial Arrangement of Molecules in Pre-Dimeric States

According to [19], H-dimers are manifested in absorption spectra as broad single shortwave bands. Such types of bands in absorption were observed for dyes I, II, III, and IV. We attempted to model the suitable configuration of all dyes using quantum chemical calculation. We optimized the geometry of all possible configurations of dimers and compared those that matched with the experimental absorption spectrum. It appeared that the most likely location of the two dye molecules in the dimers is a sandwich system wherein dipole moments are disposed, mutually, in an anti-parallel way. It is known, that planar cyanine molecules usually form dimers so that their planes are parallel. In this case, the forces of interaction reach the maximum, due to specific spacious situations [20]. As a result, the interaction between molecules in the dimer is stronger than between dyes and the surrounding matrix molecules. Therefore, optimization of the spatial geometry of dimers in a vacuum introduces an insignificant error in determining the ground state geometry parameters (we assume that interaction between guest dyes and matrix (Alq_3_) molecules can be neglected). The parameters of the four lowest electron transitions in possible dimers of the dyes studied were calculated using the TD/DFT/ WB97XD method (collected in Table 2). 

**Figure 10:**
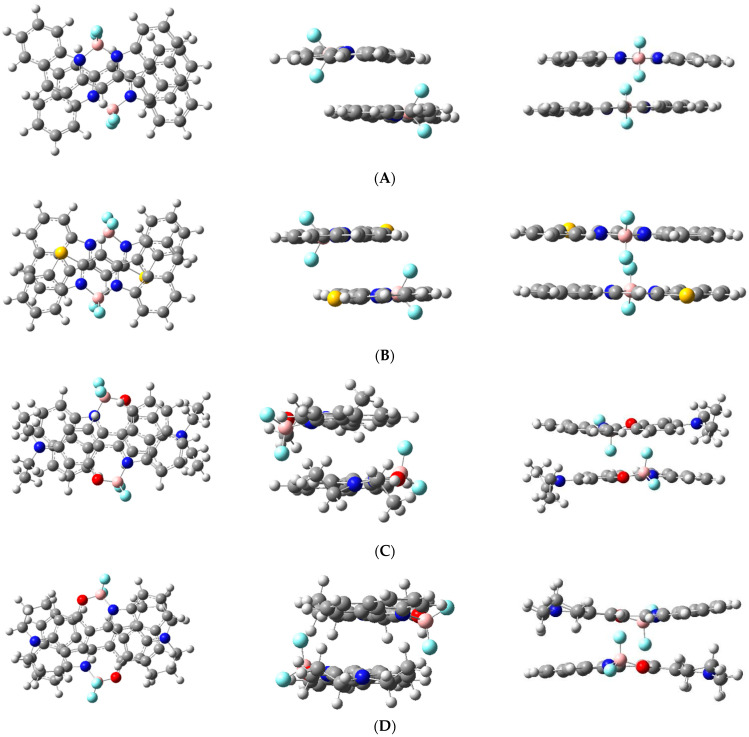
Molecular geometry of dimers of compounds I (**A**), II (**B**), III (**C**), IV (**D**), optimized by method DFT (WB97XD) in ground state.

Possible variants of the alignment of molecules in dimers were modeled and are presented in Figure 10.

**Figure 11:**
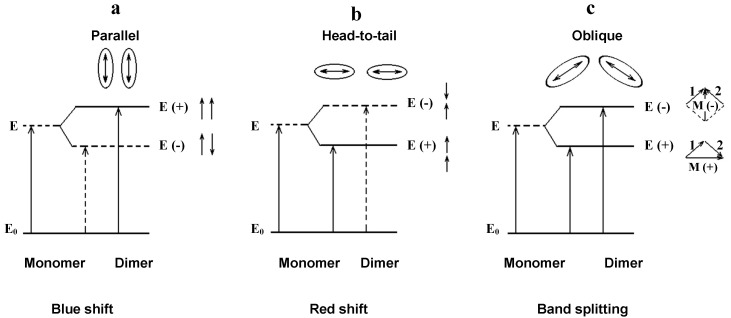
System of electronic energy levels and transitions from ground to excited state for dimers of different configurations: (**a**) “sandwich structure”, (**b**) “head to tail”, (**c**) location of molecules at some angle—”tree type”. Dotted arrows and lines indicate forbidden transition and their corresponding levels [19].

Kasha has proposed the model of “sandwich” configuration of molecules in dimers. Schemes of electronic energy levels and transitions (allowed and forbidden) for different configurations are shown in Figure 11 [19].

Scheme A corresponds to dimers of H-type (H-type dimer is the first stage of H-aggregate formation). In the case of Figure 11c, the electronic energy levels of dimers are split. With regard to the oscillator strengths of the splitting transitions, they depend on the mutual arrangement of both components; in the dimers of H-type, the longwave transition should be forbidden, whereas the probability of the shortwave transition (the oscillator strengths of the shortwave splitting transition) should increase. The calculations confirm these assumptions. The calculated parameters of the two lowest electron transitions in dimers of the studied dye are collected in Table 2.

The calculated dimer parameters (data of the first and higher excited states) were compared with data of the experimentally obtained absorption spectra (see Figure 7 and Figure 8, Table 2) of one-component films (dyes I–IV). The oscillator strengths were calculated for the first four electronic transitions and for corresponding wavelengths of the absorption bands. The calculated wavelengths for the first and second electron transitions most closely coincide with the experimentally obtained values for the parallel arrangement of molecules with almost the absence of displacement (see Figure 10).

## 4. Conclusions

In summary, we can conclude that it is most likely that the structure of one-component films of the abovementioned dyes is formed due to both monomers and dimers that do not take place in liquid solutions. The absorption, fluorescence spectra, and quantum chemical calculations indicate that these dimers are dimers of H-type. These dimers form in one-component thin films of dyes as well as in their solid solutions in Alq_3_ films. It was found that the dimers of such a type form involving guest dye molecules in the Alq_3_ matrix, starting at their concentration near 1–3%. The following increase in impurity concentration leads to a dramatically decreasing in a film’s fluorescence intensity. This effect should be taken into account in the process of luminescent materials development based on the studied dyes.

## Figures and Tables

**Table 1:** Calculated and experimental characteristic parameters of the absorption guest dyes spectra (TD/DFT/WB97XD method), where λ_calc_.—calculated wavelength of the electronic transition; f—oscillator strength.

Dye	Transition	λ_calc._, nm	f	Main Configurations
I	S_0_ → S_1_	443 (569 *)	0.67	+0.98 (HOMO → LUMO)
	S_0_ → S_2_	310	0.03	−0.44 (HOMO-2 → LUMO)
				+0.75 (HOMO → LUMO+1)
				+0.53 (HOMO → LUMO+1)
	S_0_ → S_3_	300	0.06	+0.59 (HOMO-3 → LUMO)
				+0.47 (HOMO → LUMO+1)
				+0.34 (HOMO → LUMO+3)
II	S_0_ → S_1_	422 (543*)	0.66	
+0.97 (HOMO → LUMO)	S_0_ → S_2_	305	0.11	+0.31 (HOMO-4 → LUMO)
				+0.72 (HOMO-3 → LUMO)
				−0.43 (HOMO-1 → LUMO)
	S_0_ → S_3_	276	0.15	+0.39 (HOMO-3 → LUMO)
				+0.59 (HOMO-1 → LUMO)
				+0.42 (HOMO → LUMO+1)
				+0.31 (HOMO → LUMO+2)
III	S_0_ → S_1_	429 (587 *)	0.63	+0.93 (HOMO → LUMO)
	S_0_ → S_2_	334	0.14	+0.89 (HOMO-1 → LUMO)
	S_0_ → S_3_	315	0.19	−0.31 (HOMO-4 → LUMO)+0.82 (HOMO-3 → LUMO)
IV	S_0_ → S_1_	438 (613 *)	0.63	+0.93 (HOMO → LUMO)
	S_0_ → S_2_	357	0.02	+0.92 (HOMO-1 → LUMO)
	S_0_ → S_3_	317	0.16	−0.32 (HOMO-4 → LUMO)+0.81 (HOMO-3 → LUMO)

* λ_exp._ is the wavelength of the electronic transition, determined from fluorescence and absorption spectra.

**Table 2:** Calculated and experimental characteristic parameters of the absorption dimer spectra, where calc.—calculated wavelength of the electronic transition; f—oscillator strength.

Dye	Transition	λ_calc.,_ nm	f	Main Configurations
I	S_0_ → S_1_	477 (490 *)	0.0051	−0.61 (HOMO-1→ LUMO) +0.76 (HOMO → LUMO+1)
	S_0_ → S_2_	436	0.9849	−0.38 (HOMO-1 → LUMO+1) +0.89 (HOMO → LUMO)
	S_0_ → S_3_	395	0.0085	+0.72 (HOMO-1→ LUMO) +0.57 (HOMO → LUMO+1)
	S_0_ → S_4_	388	0.1491	+0.85 (HOMO-1 → LUMO+1) +0.34 (HOMO → LUMO)
II	S_0_ → S_1_	450 (470 *)	0	+0.67 (HOMO-1 → LUMO) +0.71 (HOMO → LUMO+1)
	S_0_ → S_2_	415	0.9143	+0.33 (HOMO-1 → LUMO+1) +0.91 (HOMO → LUMO)
	S_0_ → S_3_	376	0	+0.72 (HOMO-1 → LUMO) −0.66 (HOMO → LUMO+1>)
	S_0_ → S_4_	367	0.2478	+0.92 (HOMO-1 → LUMO+1) −0.34 (HOMO → LUMO)
III	S_0_ → S_1_	453 (500 *)	0.0005	+0.60 (HOMO-1 → LUMO) +0.73 (HOMO → LUMO+1)
	S_0_ → S_2_	424	0.6811	+0.93 (HOMO → LUMO)
	S_0_ → S_3_	402	0.0229	+0.76 (HOMO-1 → LUMO) −0.60 (HOMO → LUMO+1)
	S_0_ → S_4_	390	0.4367	+0.95 (HOMO-1 → LUMO+1)
IV	S_0_ → S_1_	475 (520 *)	0.0017	−0.41 (HOMO-1 → LUMO+1) +0.80 (HOMO → LUMO>
	S_0_ → S_2_	430	0.0038	+0.56 (HOMO-1 → LUMO) −0.35 (HOMO-1 → LUMO+1) −0.50 (HOMO → LUMO) +0.52 (HOMO → LUMO+1)
	S_0_ → S_3_	424	1.0560	+ 0.69 (HOMO-1 → LUMO) +0.19 (HOMO-1 →LUMO+1) −0.59 (HOMO →LUMO+1)
	S_0_ → S_4_	409	0.0512	+0.42 (HOMO-1 →LUMO+1) +0.52 (HOMO →LUMO+1)

* λ_exp._ is the wavelength of the electronic transition, determined from absorption spectra.

## Data Availability

Not applicable.
